# Supplementary material for: A fault-intrusion-tolerant system and deadline-aware algorithm for scheduling scientific workflow in the cloud
Source: PeerJ Comput Sci. 2021 Nov 2;7:e747. doi: 10.7717/peerj-cs.747 (PMC8576554; doi:10.7717/peerj-cs.747)
Supplement: Supplemental Information 6 [file peerj-cs-07-747-s006.zip › Myproject1.1/docs/org/workflowsim/utils/ArgumentParser.html]

ArgumentParser


JavaScript is disabled on your browser.


- Overview
- Package
- Class
- Use
- Tree
- Deprecated
- Index
- Help

- Prev Class
- Next Class

- Frames
- No Frames

- All Classes

- Summary:
- Nested |
- Field |
- Constr |
- Method

- Detail:
- Field |
- Constr |
- Method


org.workflowsim.utils

## Class ArgumentParser

- java.lang.Object
- - org.workflowsim.utils.ArgumentParser

- ---

    

  ```
  public class ArgumentParser
  extends java.lang.Object
  ```

  This class implements ArgumentParser that parse arguments and set the
  parameters

  Since:
  :   WorkflowSim Toolkit 1.0

- - ### Constructor Summary

    Constructors

    Constructor and Description

    `ArgumentParser(java.lang.String[] args)`

    This method initialize a ArgumentParser object
  - ### Method Summary

    Methods

    | Modifier and Type | Method and Description |
    | --- | --- |
    | `void` | `printVersion()` |

    - ### Methods inherited from class java.lang.Object

      `clone, equals, finalize, getClass, hashCode, notify, notifyAll, toString, wait, wait, wait`

- - ### Constructor Detail


    - #### ArgumentParser

      ```
      public ArgumentParser(java.lang.String[] args)
      ```

      This method initialize a ArgumentParser object

      Parameters:
      :   `args` - argument set in WorkflowSim
  - ### Method Detail


    - #### printVersion

      ```
      public void printVersion()
      ```


- Overview
- Package
- Class
- Use
- Tree
- Deprecated
- Index
- Help

- Prev Class
- Next Class

- Frames
- No Frames

- All Classes

- Summary:
- Nested |
- Field |
- Constr |
- Method

- Detail:
- Field |
- Constr |
- Method
